# Chromatin Structure and Function in Mosquitoes

**DOI:** 10.3389/fgene.2020.602949

**Published:** 2020-12-07

**Authors:** Óscar M. Lezcano, Miriam Sánchez-Polo, José L. Ruiz, Elena Gómez-Díaz

**Affiliations:** Instituto de Parasitología y Biomedicina López-Neyra (IPBLN), Consejo Superior de Investigaciones Científicas, Granada, Spain

**Keywords:** epigenetics, ATAC-seq, ChIP-seq, vector-borne diseases, transcriptional regulation, chromatin 3D architecture

## Abstract

The principles and function of chromatin and nuclear architecture have been extensively studied in model organisms, such as *Drosophila melanogaster*. However, little is known about the role of these epigenetic processes in transcriptional regulation in other insects including mosquitoes, which are major disease vectors and a worldwide threat for human health. Some of these life-threatening diseases are malaria, which is caused by protozoan parasites of the genus *Plasmodium* and transmitted by *Anopheles* mosquitoes; dengue fever, which is caused by an arbovirus mainly transmitted by *Aedes aegypti*; and West Nile fever, which is caused by an arbovirus transmitted by *Culex* spp. In this contribution, we review what is known about chromatin-associated mechanisms and the 3D genome structure in various mosquito vectors, including *Anopheles*, *Aedes*, and *Culex* spp. We also discuss the similarities between epigenetic mechanisms in mosquitoes and the model organism *Drosophila melanogaster*, and advocate that the field could benefit from the cross-application of state-of-the-art functional genomic technologies that are well-developed in the fruit fly. Uncovering the mosquito regulatory genome can lead to the discovery of unique regulatory networks associated with the parasitic life-style of these insects. It is also critical to understand the molecular interactions between the vectors and the pathogens that they transmit, which could hold the key to major breakthroughs on the fight against mosquito-borne diseases. Finally, it is clear that epigenetic mechanisms controlling mosquito environmental plasticity and evolvability are also of utmost importance, particularly in the current context of globalization and climate change.

## Introduction

In recent years, there has been an explosive growth of studies focused on the multiple layers of chromatin organization in metazoans and their function controlling genome activity (Sexton and Cavalli, 2015; Bonev and Cavalli, 2016). These studies have revealed a major complexity and plasticity of the 3D genome structure, which must be robust in time as well as flexible enough to allow for effective responses to environmental constraints. Yet, most evidence is still restricted to laboratory conditions and model organisms, such as the fruit fly *Drosophila melanogaster* (Sexton et al., 2012; Rowley et al., 2017).

Mosquitoes, such as *Anopheles*, *Aedes*, and *Culex* spp., are a major global health concern because they are vectors of life-threatening diseases. These include malaria, dengue, filariasis, or Zika, West Nile, and Chikungunya fevers, which cause millions of deaths yearly in Africa, Asia, and South America. Despite the fact that there have been considerable advances in the field of mosquito genomics, little is known about their regulatory genome and the epigenetic regulation of gene expression, in particular in the context of an infection (Shaw and Catteruccia, 2019; Compton et al., 2020b). These gaps of knowledge are critical, considering the natural variability in their transmission potential (i.e., vector competence, which is dependent on environmental factors), and their ability to adapt rapidly to new environments. Notably, the evolution and spread of insecticide-resistant mosquitoes are rendering current approaches to fight disease useless. This, together with the increasing ineffectiveness of available drugs against the pathogens, has promoted the development of advanced gene editing strategies for vector and disease control (Shaw and Catteruccia, 2019; Li et al., 2020). While harboring great potential, these technologies require a comprehensive knowledge about mechanisms of transcriptional regulation in the targeted organisms, as well as a detailed characterization of the gene regulatory networks operating at different developmental stages and in different tissues.

The focus of this review is to provide an overview of studies that have begun to describe the mechanisms of transcriptional regulation in vector mosquitoes, including 3D genome organization, chromatin structure, and epigenetic mechanisms, mainly in *Anopheles*, which is the most intensively studied genus, but also in *Aedes* and *Culex* spp. We also aim to discuss the gaps that remain unexplored in these insects, in particular, how the regulatory genome changes dynamically through development and which are the epigenetic mechanisms underlying regulatory plasticity in response to external stimuli. Finally, we advocate that such new insights into mosquito biology can be revolutionary in the field and are fundamental to overcome the plasticity and adaptation of these deadly insects to environmental heterogeneity in the efforts to eradicate old and novel infectious diseases.

## 3D Genome Organization

**FIGURE 1:**
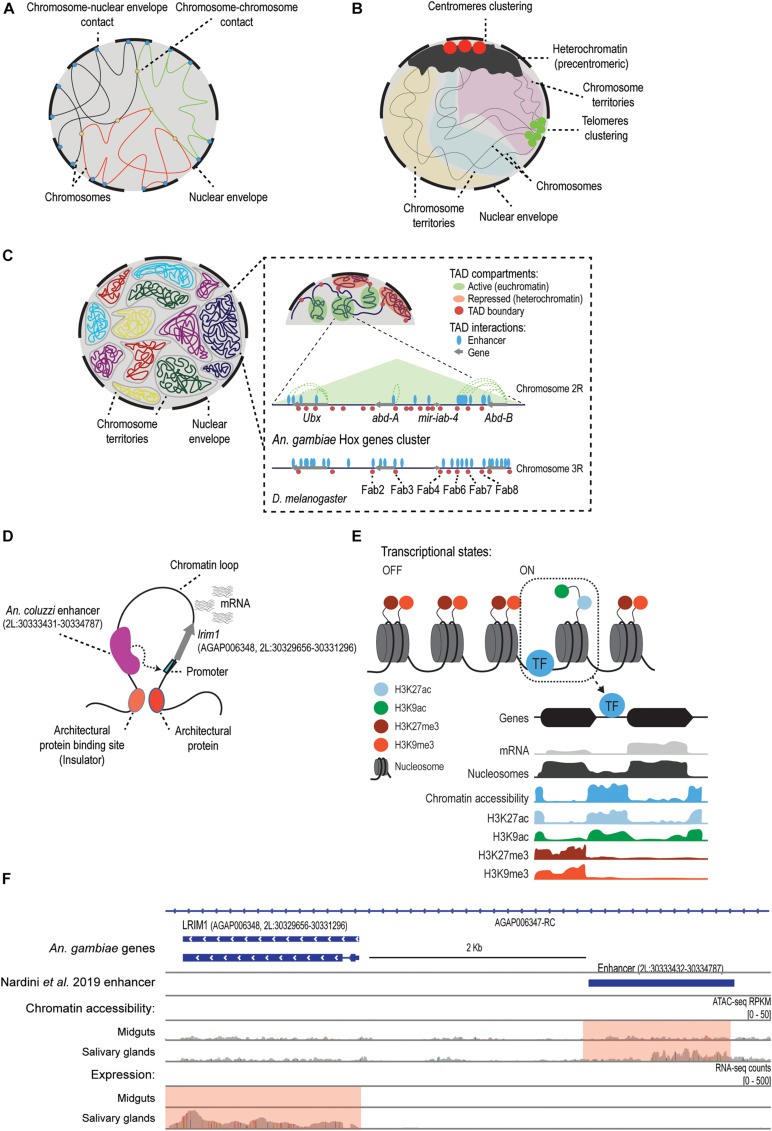
The regulatory genome of mosquitoes. **(A)** In *Anopheles* mosquitoes, as previously described for *Drosophila* (Moretti et al., 2020), the attachment of the chromatin fiber to the nuclear envelope and lamina contributes to the organization and functional 3D structure of the genome, and it determines the contact frequencies between and within chromosomes (George et al., 2020; Lukyanchikova et al., 2020). **(B)** The Rabl-like configuration described in *Anopheles* spp. (would contribute to the reduction of DNA entanglement by attaching heterochromatic centromeres and telomeres at opposite poles of the cell (George et al., 2020; Lukyanchikova et al., 2020). Panels A and B are partly adapted from Figure 4B in Lukyanchikova et al. (2020). **(C)** A representation of Topologically Associating Domains (TADs). First panel shows chromosomal territories inside the nucleus. Panels with higher magnification show the regulatory landscape (not to scale) reported by Ahanger et al. (2013), around the *An. gambiae* Hox genes cluster known as the bithorax complex, which is conserved in *D. melanogaster* (Ahanger et al., 2013). The name of some known insulators in *Drosophila* that seem to be conserved in *An. gambiae* are included. **(D)** Model of transcriptional regulation based on the extrusion of chromatin loops, which allows for the interaction between cis-regulatory elements (i.e., enhancers) and their target promoters. One example is the *lrim1* gene and its enhancer region, which was characterized using STARR-seq in *An. coluzzi* (Nardini et al., 2019). How the interaction between the enhancer and the *lrim1* promoter alters the chromatin structure and the transcriptional status of the gene remains to be studied. **(E)** Model of the mechanisms of transcriptional regulation in mosquitoes. Chromatin structure is dynamic during development or in response to external stimuli. Post-translational modifications of histones control transcription by recruiting chromatin modifiers or by modulating the accessibility of regulatory proteins. Transcription factors bind to regulatory sequences in accessible regions and activate or repress transcription. Certain histone modifications, such as H3K27ac, are enriched at accessible regions and active genes, whereas others such as H3K27me3 are associated to gene silencing and heterochromatin (Gómez-Díaz et al., 2014; Ruiz et al., 2019). Adapted from Ruiz and Gómez-Díaz (2019). **(F)** Snapshot of the genomic region in *An. gambiae* containing the *lrim1* gene and its enhancer region located 2 Kb upstream. This enhancer was originally described and validated in *An. coluzzi* by Nardini et al. (2019). The chromatin accessibility and gene expression profiles obtained for this region in *An. gambiae* (Ruiz et al., 2020) are included. According to Ruiz et al. (2020), *lrim1* is differentially expressed between midguts and salivary glands, and this is associated with differential chromatin accessibility at the enhancer region (pink box). Adapted from Ruiz et al. (2020).)

The genome organization within the nucleus has different components, such as the distribution of chromosomal territories, the intra- and inter-chromosomal contacts, and the attachment with the nuclear envelope (Deng and Blobel, 2014; Misteli, 2020; Figures 1A,B). Importantly, the spatial configuration of the genome has been shown to play a role in orchestrating tissue-, cell-, and stage-specific transcriptional regulation during development and in differentiation, pathogenesis, as well as in response to external stimuli (Cremer et al., 2014; Belyaeva et al., 2017; Cattoni et al., 2017; Rowley et al., 2017; Finn and Misteli, 2019; Ing-Simmons et al., 2020). Studies on *Drosophila* have been a rich source of information about the way the metazoan genome is organized and compartmentalized at the 3D level (Sexton et al., 2012; Rowley et al., 2017) and the functional consequences of changes in genome topology, with many general principles of *Drosophila* chromatin organization and dynamics being evolutionary conserved (Rowley et al., 2017). Similar studies in disease-vector mosquitoes have just started to emerge (Sharakhov and Sharakhova, 2015; Wiegmann and Richards, 2018; Li F. et al., 2019; Ruzzante et al., 2019; Compton et al., 2020b). Traditional physical mapping approaches, such as FISH and optical mapping, have been applied in *Ae. aegypti* (Sharakhova et al., 2011; Timoshevskiy et al., 2013, 2014), *Cx. quinquefasciatus* (Naumenko et al., 2015), *Cx. tarsalis* (Little, 2020), and several *Anopheles* species (Cornel and Collins, 2000; Sharakhov et al., 2002, 2004, 2016; Sharakhova et al., 2010; George et al., 2010, 2020; Xia et al., 2010; Jiang et al., 2014; Artemov et al., 2015, 2017, 2018; Neafsey et al., 2015; Wei et al., 2017; Lukyanchikova et al., 2020; Waterhouse et al., 2020), and they contributed not only to the improvement of the genomes annotation, by assessing the ordering and orientation of the contigs and scaffolds, but also to the study of the organization of centromeres in different cell types (Sharakhova et al., 2019; Lukyanchikova et al., 2020). The advantage of these methods is that they make genome mapping more generalizable to non-model mosquitoes (Sharakhova et al., 2019). Hi-C is a high-throughput sequencing technique based on chromosome conformation capture that aims to study the 3D genome folding and chromatin interactions by measuring the frequency of contacts between loci (van Berkum et al., 2010). Until recently, the application of Hi-C had been limited to the improvement of the genome assembly of several mosquito species: *Culex quinquefasciatus* (Dudchenko et al., 2017), *Aedes aegypti* (Dudchenko et al., 2017; Matthews et al., 2018), *Ae. albopictus* (Palatini et al., 2020), *Anopheles albimanus* (Compton et al., 2020a), *An. funestus* (Ghurye et al., 2019a,b), *An. stephensi* (Chakraborty et al., 2020), *An. coluzzi* (Zamyatin et al., 2020), and *An. arabiensis* (Zamyatin et al., 2020). Lukyanchikova et al. (2020) recently applied Hi-C to map genome-wide chromatin contacts in five *Anopheles* species (*An. coluzzi*, *An. merus*, *An. stephensi, An. atroparvus*, and *An. albimanus*), revealing unique features of their 3D genome structures. For example, this work delineated five scaffolds that correspond to known chromosomes (X, 2R, 2L, 3R, 3L) and revealed several regions characterized by butterfly contact patterns, that is, splits between chromatin blocks in the Hi-C map that are typically associated with chromosomal rearrangements, which in the case of *Anopheles* correspond to known balanced inversions (Corbett-Detig et al., 2019; Lukyanchikova et al., 2020).

Topologically Associating Domains (TADs) are considered to be the basic units in the genome structure and function (Dixon et al., 2012; Szabo et al., 2018; Misteli, 2020). In mosquitoes, as in other metazoans, these TADs correspond to regions of the genome with a high degree of contacts that reflect the regulatory events that are taking place (Dixon et al., 2012; Cubenas-Potts and Corces, 2015; Chen et al., 2018; Figures 1C,D). As expected based on the TADs found in *Drosophila* (Eagen et al., 2015; Ulianov et al., 2016), *Anopheles* spp. chromosomes appear to be partitioned into two non-overlapping compartments: euchromatin (A-compartments) and heterochromatin (B-compartments). TADs found in A-compartments tend to be smaller and are associated with active gene expression, while longer TADs in B-compartments are gene-poor and correspond to regions with low levels of gene expression (Lukyanchikova et al., 2020; Figure 1C). The Hi-C study by Lukyanchikova et al. (2020) defined 200–400 Kb as the characteristic TAD length in *Anopheles*, which is similar to the typical length that they defined for *D. melanogaster* TADs, and smaller than the TAD length reported in *Ae. aegypti*, of around 500–800 Kb. In addition, by studying chromatin contact probability they found that, as expected, contact frequency decays as a function of genomic distance between chromatin loci, but this happens non-uniformly (i.e., in two different decay phases, with the second phase falling sharply), meaning that short-range interactions occur more frequently. Accordingly, the vast majority of *Anopheles* loops are less than 1 Mb-sized, but they also found a number of long chromatin loops (2–6 loops of dozens of megabases, up to a distance of 31 Mb) that appear to be evolutionary conserved between *Anopheles* spp. (Lukyanchikova et al., 2020). Compared to *Drosophila*, these Mb-scaled loops represent extremely long-range interaction contacts (Lukyanchikova et al., 2020). Strikingly, they do not appear to be associated with the clustering of active genes and also display low levels of H3K27me3 enrichment, which indicates that neither correspond to Polycomb-mediated loops. These findings have led the authors to suggest new principles of 3D genome organization in *Anopheles* spp. With regards to the functionality of these TADs in mosquitoes, we know relatively little. Despite some controversy on whether genome conformation or transcription is more important to gene control (Ing-Simmons et al., 2020), it is well-established that TAD structure plays a role in transcriptional regulation (Beagan and Phillips-Cremins, 2020). Several studies in *Drosophila* and other eukaryotes have shown that disruption of TAD boundaries and TAD rearrangements alter enhancer-promoter interactions and dysregulate gene expression (Liao et al., 2020). This has led to suggestions that TAD structure should be highly evolutionary constrained between related species, for example, across dipterans. Indeed, TADs have been shown to be conserved across *Drosophila* species (Renschler et al., 2019; Liao et al., 2020; Torosin et al., 2020). However, the differences in TAD length reported above between *Anopheles* spp., *Ae. aegypti*, and *D. melanogaster* suggest some of these TADs could be mosquito species- or genus-specific. Further work profiling TADs in different mosquito species and tissues, combined with epigenomic and transcriptomic data, for example, by using HiChIP experiments, could support the conclusion that these variable patterns in mosquitoes reflect different cis-regulatory mechanisms.

Architectural proteins are important regulators of the 3D genome organization in metazoans that contribute to the establishment of interactions between regulatory elements across multiple spatial scales (Gomez-Diaz and Corces, 2014; Misteli, 2020). Different protein combinations are present in the genomes at specific binding sites, generally at TAD boundaries, and they show varied roles in genome organization and function. For instance, they may have an insulator function preventing unspecific enhancer-promoter communication, and/or mediate the interaction with the proper target promoter by chromatin looping (Gomez-Diaz and Corces, 2014). Five insulator proteins have been found in *D. melanogaster*, but only CTCF has orthologs in other phyla (Ong and Corces, 2014; Schoborg and Labrador, 2014). Interestingly, other *Drosophila* architectural proteins, such as Su(Hw), CP190, and GAF, also have orthologs in mosquito genomes, including *Anopheles* spp., *Ae. aegypti*, and *Cx. quinquefasciatus* (Kriventseva et al., 2019; Thurmond et al., 2019). Initial studies about CTCF in *An. gambiae* and *Ae. aegypti* also reported that the protein is constitutively expressed and binds to known insulator sequences (Gray and Coates, 2005). Their role is further supported by the findings regarding the Hox complex of *Drosophila*, which contains several architectural proteins binding sites at the genes boundaries that appears to be conserved in *An. gambiae* (Ahanger et al., 2013; Figure 1C). Some boundary elements in *An. gambiae* were also functionally validated in enhancer-blocking assays in transgenic flies, demonstrating that they function as insulators to the same extent as other endogenous architectural proteins in the fly, such as Fab-7 and Fab-8 (Figure 1C; Ahanger et al., 2013). Exploring which are the regulatory binding sequences and the architectural proteins controlling TADs function in mosquitoes will likely contribute to a better understanding of the molecular machinery regulating genome structure and function.

The spatial organization of the genome within the nucleus is also known to be controlled by chromatin interactions with the nuclear envelope (Cavalli and Misteli, 2013; Figure 1A). In *Anopheles* spp., the nuclear envelope attachment has been proposed to reduce topological entanglement of chromosomes (George et al., 2020; Lukyanchikova et al., 2020), and Hi-C data supports a Rabl-like configuration, as in *Drosophila* (Moretti et al., 2020). This is characterized by the clustering of centromeres and telomeres to the nuclear envelope at opposite poles of the nucleus, and the more elongated shape of the chromosome territories (Wilkie et al., 1999; Lukyanchikova et al., 2020; Figure 1B). However, when comparing the results of experiments in *Anopheles* spp. embryos with those in adults of *An. merus*, the Rabl-like configuration was less pronounced in the adult tissues (Lukyanchikova et al., 2020). Another study using confocal microscopy and FISH in three *Anopheles* spp. (*An. gambiae, An. coluzzi*, and *An. merus*) (George et al., 2020) found chromosome territories that appeared ellipsoidal in shape, not spherical, as in mammals (Khalil et al., 2007; Sehgal et al., 2014). This is important because these various shapes can influence the distance and frequencies of the spatial interactions in the genome (Lukyanchikova et al., 2020). Given these incongruities, it would be necessary to study the dynamics of this configuration considering different species tissues, and developmental stages.

Taken together, the studies above have been pioneers in the characterization of the 3D genome organization in mosquitoes and provided first insights into how it relates to transcriptional regulation. However, a considerable amount of work is still needed to unravel fundamental processes such as TAD formation, maintenance and function, the role of architectural proteins in mediating chromatin looping, or the formation and function of Polycomb and trithorax complexes.

## Chromatin Structure and Regulation of Gene Expression

Together with the spatial genome organization within the nucleus, the local structure of chromatin also contributes to transcriptional regulation. Post-translational modifications of histone tails, such as methylation, acetylation, and phosphorylation, can significantly alter chromatin accessibility and protein binding at regulatory regions, and this in turn affects gene expression (Sharakhov and Sharakhova, 2015; Figure 1E). The histone modifications landscape seems to be generally well-conserved between *Drosophila* and *Anopheles* spp. (Gómez-Díaz et al., 2014; Ruiz et al., 2019; Ruiz et al., 2020). Unfortunately, no available data exists for mosquito species of the genera *Aedes* and *Culex*. In the case of *An. gambiae*, Gómez-Díaz et al. (2014) profiled the transcriptome by RNA-seq and the global occupancy of H3K27me3 and H3K27ac histone modifications by Chromatin Immunoprecipitation sequencing (ChIP-seq). This allowed the identification of various chromatin states that correlate with tissue-specific functions, and resemble those previously found in *D. melanogaster* (Kharchenko et al., 2011; Negre et al., 2011). For instance, the authors reported mutually exclusive distribution of H3K27ac and H3K27me3: H3K27ac enrichment was found downstream from transcription start sites (TSSs) of active genes, while H3K27me3 filled broader intergenic regions and appeared associated with heterochromatic clusters of silenced genes, which correspond to *Drosophila* Polycomb-associated domains. Another study interrogated the dynamics of histone modification patterns in *An. gambiae* in the context of an infection by the malaria parasite *Plasmodium falciparum* (Ruiz et al., 2019). In particular, the authors examined changes in the abundance of various active and repressor histone modifications (H3K9ac, H3K27ac, H3K4me3, and H3K9me3) in infected and uninfected *An. gambiae* mosquitoes. This comparison allowed the identification of regions with changing histone modifications profiles that annotated to malaria-responsive genes involved in immune functions, such as antimicrobial peptides, CLIP proteases, or members of the melanization and complement systems. Overall, these studies have given an initial view of the histone modifications landscape in malaria mosquito vectors and their implications in chromatin regulation, providing evidence that they play a key role in directing transcriptional responses to environmental stimuli, such as a parasitic infection. Yet, a precise characterization of the underlying mechanisms is still lacking, including the writers and erasers that modulate histone modifications dynamics and the readers that can interpret them. Whether these epigenetic patterns are evolutionary conserved in other mosquito species also requires further investigation.

Another area in the mosquito field that is accumulating new evidence is the characterization and mapping of cis-regulatory elements (CREs), i.e., regions of non-coding DNA that are involved in the transcriptional regulation of their neighboring genes (Li et al., 2011; Voss and Hager, 2014; Reiter et al., 2017). These regulatory elements include sequences such as promoters, enhancers, and silencers. Thousands of CREs have been discovered in *Drosophila* over the last decades (Gallo et al., 2006, 2011; Halfon et al., 2008; Kvon et al., 2014; Slattery et al., 2014; Vizcaya-Molina et al., 2018; Rivera et al., 2019; Gao and Qian, 2020), and this knowledge has enabled some progress about their existence and function in various mosquito species, including *An. gambiae*, *Ae. aegypti*, and *Cx. quinquefasciatus* (Sieglaff et al., 2009; Ahanger et al., 2013; Kazemian et al., 2014). While there have been many studies characterizing the regulatory sequences of specific genes, for example, for the *sog* gene controlling the dorsal-ventral patterning in *Ae. aegypti* (Behura et al., 2016; Suryamohan et al., 2016; Mysore et al., 2018), and *An. gambiae* (Goltsev et al., 2007; Cande et al., 2009; Kazemian et al., 2014), or the cytochrome P450 *Cyp9m10* gene involved in insecticide resistance in *Cx. quinquefasciatus* (Itokawa et al., 2011; Wilding et al., 2012), the vast majority of mosquito CREs reported to date are computational predictions and/or still lack experimental verification (Sieglaff et al., 2009; O’Brochta et al., 2012; Ahanger et al., 2013; Kazemian et al., 2014; Price et al., 2015; Behura et al., 2016; Perez-Zamorano et al., 2017; Mysore et al., 2018; Nardini et al., 2019; Ruiz et al., 2019, 2020; Brody et al., 2020). The application of state-of-the-art methods for the genome-wide profiling of chromatin accessibility that allow the identification of functional CREs is therefore crucial. The first studies in this area used Formaldehyde-Assisted Isolation of Regulatory Elements (FAIRE-seq) (Giresi et al., 2007) for the discovery of active regulatory sequences in the genomes of *An. gambiae* (Perez-Zamorano et al., 2017) and *Ae. aegypti* (Behura et al., 2016; Mysore et al., 2018). The study by Mysore et al. (2018) reported a set of CREs driving tissue-specific gene expression in neurons of the olfactory system of *Ae. aegypti*. For example, they studied some CREs that are adjacent to odorant receptor (*Or*) genes and TFs that regulate *Or* expression in the adult antennae, such as *orco*, *Or1*, *Or8*, and *fru*, which also drove transgene expression in *Drosophila*. On the other hand, the study by Behura et al. (2016) also reported a set of active regulatory sequences in whole *Ae. aegypti* embryos, which were functional in transgenic *Drosophila* reporter assays for multiple tissues. While these studies represent the first chromatin accessibility maps in mosquitoes, the FAIRE-seq technique displays low resolution and limited accuracy in identifying DNA-protein binding events. In contrast, the Assay for Transposase Accessible Chromatin with sequencing (ATAC-seq) has emerged as one of the most powerful approaches for genome-wide chromatin accessibility profiling, allowing a more precise identification of regulatory regions, such as promoters, TSSs, or enhancers, as well as the prediction of TF binding events (Buenrostro et al., 2013; Karabacak Calviello et al., 2019; Li Z. et al., 2019; Li, 2020). A recent study using ATAC-seq in combination with RNA-seq in different *An. gambiae* tissues (Ruiz et al., 2020) revealed a precise genome-wide map of CREs involved in the control of tissue-specific gene expression and predicted *in vivo* binding sites of relevant transcription factors. Results showed that a great portion of regulatory sites are located at introns, followed by those annotated to TSSs and exons, suggesting a predominant role of intragenic CREs in mosquito transcriptional regulation. They also combined the ATAC-seq data and a homology-based sequence prediction from *Drosophila* to identify CTCF-like binding sites that could function as insulators. Furthermore, by comparing chromatin accessibility and transcriptional profiles at different tissues, this study allowed for the functional characterization of hundreds of enhancers and TSSs, some of which appear to control genes involved in *Anopheles* responses against *Plasmodium* infection (Figure 1F). This data is of great potential in the pursuit of new vector-control and anti-malaria strategies. Future work applying gene editing techniques to confirm the novel *An. gambiae* enhancers, together with ChIP-seq experiments of the predicted TFs, would be valuable tools in further validating these CREs. These results also open the door to similar ATAC-seq experiments in other mosquitoes that are vectors of major diseases including *Aedes* and *Culex* spp.

## Additional Layers of Epigenetic Regulation

One basic epigenetic mechanism that mediates local chromatin structure and gene activity in metazoans is DNA methylation, which involves the covalent transfer of a methyl group to the cytosines by the action of several DNA methyltransferases (Kumar et al., 2018). The methylated state alters gene expression by recruiting repressors or by inhibiting the binding of transcription factors. However, dipterans belonging to the “Dnmt2 only” organisms do not contain any of the canonical DNA methyltransferases (Dnmt1 and Dnmt3) (Krauss and Reuter, 2011; Bewick et al., 2017; Provataris et al., 2018; Lewis et al., 2020). The remaining Dnmt2 does not appear to methylate DNA, but instead it methylates tRNA (Goll et al., 2006; Bewick et al., 2017). Despite some authors arguing that Dnmt2 may serve as a methyltransferase of both specific DNA and tRNA targets (Krauss and Reuter, 2011), the level of 5-methylcytosine found in *D. melanogaster* (<0.5%), is very low compared to the levels in other metazoans and seems to be restricted to embryonic development (Gowher et al., 2000; Lyko et al., 2000; Marhold et al., 2004; Phalke et al., 2009; Krauss and Reuter, 2011; Zhang et al., 2015; Bewick et al., 2017). In *An. gambiae*, initial studies reported 0.49% of methylation based on slot blots and capillary electrophoresis (Marhold et al., 2004). More recently, there have been other studies that analyzed DNA methylation in various mosquito species using whole-genome bisulfite sequencing (Falckenhayn et al., 2016; Bewick et al., 2017). Falckenhayn et al. (2016) reported the lack of DNA methylation and known DNA methyltransferases in *Ae. aegypti*. Bewick et al. (2017) analyzed several dipterans, including *Ae. aegypti*, *Ae. albopictus*, *Cx. quinquefasciatus*, *An. gambiae*, and *D. melanogaster*, showing genome-wide methylation levels very close to 0%. Contrarily, DNA methylation was present in all other orders of insects with variable levels reaching 10–15%. The low levels of DNA methylation in dipterans are consistent with the proposed residual role of Dnmt2 as RNA methyltransferase. However, the functional significance of Dnmt2-mediated methylation is being challenged in recent years (Takayama et al., 2014; Lewis et al., 2020). For example, this mechanism has been suggested to be involved in immune responses in *D. melanogaster* (Durdevic and Schaefer, 2013; Bhattacharya et al., 2020), and in this species the encoding gene has been shown to display positive selection signatures (Bhattacharya et al., 2020). In mosquitoes, Ye et al. (2013) showed changes in the methylation patterns of *Ae. aegypti* linked to *Wolbachia* infection, but the link with Dnmt2 remained unclear. More recently, Claudio-Piedras et al. (2019) reported that the pharmacological inhibition of the methyltransferase activity (Dnmt2) impacted *An. albimanus* larval viability and susceptibility to the malaria parasite *Plasmodium berghei*, and these changes in the phenotype were accompanied with changes in global levels of DNA methylation detected by immunodetection (dot blot). Further, using an *in silico* analysis, this study identified components of a methylation system in *An. albimanus*, including the genes *mbd*, *tet2*, and *dnmt2.* Together, these results suggest a functional role of Dnmt2-mediated methylation in the mosquito response to infection, but this study has some caveats. First, the precise relationship between the decitabine and azacytidine treatments with genome-wide transcriptional regulation was not assessed (Claudio-Piedras et al., 2019). Second, the systemic cytotoxic effects of these treatments are known from studies in other organisms, including *Drosophila* (Katz, 1985; Cunha et al., 2002). In these studies, the effects and toxicity of the drugs have been shown to be variable across developmental stages, tissues, and cell types (Laurent et al., 2010; Foret et al., 2012; Rasmussen et al., 2016; Cook et al., 2019) and also depend on the drug dosage (Yang et al., 2006; Cook et al., 2019). The study by Claudio-Piedras et al. (2019) did not report toxicity in the mosquito *An. albimanus* using a concentration of 50 μM. Cunha et al. (2002) tested a range of concentrations from 25 to 250 μM in *D. melanogaster*, showing global mutagenic activity independently of the dose. The mutagenic effects of this drug on DNA, which is the result of the formation of the Dnmt2-nucleoside adduct and the subsequent repair, is expected to be proportional to the number of cytosines in the DNA that are targeted by Dnmt2. Therefore, such a global toxicity does not seem to agree with the Diptera’s low Dnmt2 activity, and instead a marginal effect would be expected (Stresemann and Lyko, 2008; Cook et al., 2019). Beyond these initial observations, to validate the function of DNA methylation in mosquitoes, it will be necessary to silence the Dnmt2 enzyme, with iRNA or CRISPR/cas9, and to study the genome-wide effects at the level of DNA methylation, using bisulfite sequencing, and at the level of gene expression by RNA-seq.

Another field that has experienced considerable advances in recent years is the study of mosquito non-coding RNAs, particularly micro-RNAs (miRNAs). Whether these RNA species can be considered truly epigenetic is still the subject of intense debate, but it is now clear that they play important functions in several chromatin-associated processes, including: RNA directed gene silencing, chemical (i.e., Xist) and structural changes to chromatin (i.e., enhancer RNAs), and mediation of the regulation of gene promoters (Kurokawa et al., 2009; Wang et al., 2011; Lam et al., 2014; Maleszka, 2016; Moutinho and Esteller, 2017). In mosquitoes, their role in the regulation of gene expression at the transcriptional and post-transcriptional levels has been shown to contribute to physiological and immune pathways, and to affect processes such as development, metabolism, blood digestion, host-pathogen interactions, and insecticide resistance (Li et al., 2009; Bryant et al., 2010; Liu et al., 2014; Lucas et al., 2015; Tian et al., 2016; Zhang et al., 2016; Feng et al., 2018; Fu et al., 2020). The type and abundance of miRNAs vary across mosquito species, between sexes, stages, tissues, and organs (Feng et al., 2018), with some being specific and evolutionary conserved (Li et al., 2009; Skalsky et al., 2010). Regarding their mechanisms of action, a recent study used CLEAR-CLIP to build miRNA-mRNA interaction networks during egg maturation in female *An. gambiae* (Fu et al., 2020) and revealed multi-target interactions, so some miRNAs may use different regions to bind several targets without changing their sequence. This implies a considerable expansion of the miRNA target repertoire, allowing mosquitoes to regulate a more diverse array of target genes in a tissue- and stage-specific manner. Despite this diversity, few miRNAs have been functionally validated. This is the case of the ovarian-specific miRNA-309, whose silencing in *Ae. aegypti* led to repression of genes involved in development, sex determination, and chromatin regulation (Zhang et al., 2016). Other studies have focused on miRNAs involved in the regulation of mosquito-pathogen interactions. In particular, four miRNAs have been shown to be altered upon *An. gambiae* infection by the rodent malaria parasite *P. berghei*, whereas the silencing of Dicer1 and Ago1 increased parasite survival (Winter et al., 2007). Another case is miR-2940, which has been reported to be upregulated in *Wolbachia-* and arbovirus-infected *Ae. aegypti* and *Ae. albopictus* mosquitoes (Skalsky et al., 2010; Hussain et al., 2011, 2013; Zhang et al., 2013; Slonchak et al., 2014). This miRNA upregulates the metalloprotease m41 FtsH, which is required for efficient West Nile Virus replication (Slonchak et al., 2014) and *Wolbachia* infection (Hussain et al., 2011), and it also downregulates the *dnmt2* gene, which is required for dengue replication (Zhang et al., 2013). Similarly, *Ae. aegypti* miR-375 may play a role in dengue virus infection by controlling the immune function of the transcription factors cactus and REL1 (Hussain et al., 2013), and miR-92 and miR-989 were differentially expressed in *Cx. quinquefasciatus* after West Nile Virus experimental infections (Skalsky et al., 2010). Altogether, the studies above illustrate well the implications and relevance of the study of RNA–chromatin interactions in mosquitoes, an area that calls for future research.

## Concluding Remarks

Collectively, the evidence discussed in this review points to multiple epigenetic mechanisms controlling transcriptional regulation during development and the dynamic responses of mosquitoes to the environment. The principles governing the chromatin structure and 3D organization of the genome appear to be mostly conserved between the few mosquito species studied, and the patterns are in most cases shared with *Drosophila*. There are, however, some exceptions that remain to be confirmed, for example, the existence of Polycomb-independent chromatin looping mechanisms or the still controversial role of DNA methylation. Areas for further work include the functional validation and characterization of the recently described enhancer maps in different mosquito tissues and stages, and the identification of the molecular components and mechanisms regulating the architecture and function of the mosquito genome. These advancements would not only serve to gain new knowledge on the biology of these organisms, but they could also inform novel mosquito control strategies that block disease transmission.

## Author Contributions

EG-D, JLR, MS-P, and ÓL wrote the manuscript. JLR made the figure. All authors read and approved the final manuscript.

## Conflict of Interest

The authors declare that the research was conducted in the absence of any commercial or financial relationships that could be construed as a potential conflict of interest.
